# Severe odontogenic infections: a 5-year review of a major referral hospital in Ghana

**DOI:** 10.11604/pamj.2019.32.71.17698

**Published:** 2019-02-12

**Authors:** Paa-Kwesi Blankson, Grace Parkins, Matthew Owusu Boamah, Alhassan Emil Abdulai, Abdul-Majeed Ahmed, Sarah Bondorin, Isaac Nuamah

**Affiliations:** 1 Department of Oral & Maxillofacial Surgery, Korle-Bu Teaching Hospital, Accra, Ghana; 2 School of Medicine & Dentistry, College of Health Sciences, University of Ghana, Accra, Ghana

**Keywords:** Odontogenic, infection, cumulative incidence, cellulitis

## Abstract

**Introduction:**

Odontogenic infections are fairly common in healthcare settings. However, late presentations such as Ludwig's angina, facial cellulitis, necrotizing cervical fasciitis (NCF), among others could lead to mortality. In view of suggestions that the occurrence of severe, near-fatal odontogenic infections is declining, this study set out to determine the incidence of such severe odontogenic infections over the past 5 years at the Korle-Bu Teaching Hospital, a major referral centre in Ghana.

**Methods:**

A retrospective review was done, involving all patients with severe odontogenic infection, thereby requiring admission, per stated criteria at the Department of Oral and Maxillofacial Surgery (Dental clinic), Korle-Bu Teaching Hospital, in the period between July 2012 and July 2017. The cumulative incidence for the respective years were then computed for the years of review.

**Results:**

A total of 243 patients were included in the study. This consisted of 121 males and 122 females, with an average age of 42.9 years (SD = 16.6), ranging from 18 months to 91 years. Incidence proportions for the years of the review were 8.2, 8.9, 17.7, 17.9 and 27.7 people per 1000 cases of tooth-related infections for the respective years. With a fatality rate of 5.8%, the incidence of odontogenic infections among patients attending the outpatient Dental clinic of the hospital is 40.3%, while that of dentoalveolar abscess is 6.2%. Ludwig's angina was the commonest (52%) form of presentation of spreading odontogenic infection.

**Conclusion:**

This study highlights the importance of persisting severe, near-fatal odontogenic infections in Ghana. Not only is there a need to assess the public, professional and institutional strategies to management, but for more evidence-based studies in our local setting to aid in management.

## Introduction

Odontogenic infections, in their severe forms have generally been considered to be decreasing in occurrence [1, 2], with many attributed reasons such as availability of antimicrobials [1], advancements in healthcare delivery and general improvement in oral hygiene, with subsequent reduction in mortality [3]. Infections of dental origin in themselves are fairly common, with some authors suggesting it to account for a proportionate amount of antibiotic prescription [4]. However, when not controlled, these could spread into anatomically associated spaces of the maxillofacial and cervical region, thus risking myriad possible complications including respiratory obstruction, Ludwig's angina, facial cellulitis, deep neck abscesses, NCF, aspiration pneumonia, septicaemia, descending mediastinitis, brain abscess, thoracic empyema, endocarditis, pericarditis, pleuropulmonary suppuration, pneumothorax, abscess of the carotid sheath and jugular thrombophlebitis, hematogenous dissemination to distant organs, and coagulation abnormalities ranging from thrombocytopenia to a fulminant state of disseminated intravascular coagulation (DIC), which could all eventually result in mortality [2, 3, 5, 6]. Suggested guidelines used by the Maxillofacial unit, recommends hospital admission of patients with severe odontogenic infections, characterized by one or more of the following signs/symptoms; rapidly progressing infection, involvement of multiple fascial spaces or high-risk spaces such as the pterigo-mandibular space, unrelenting fever or signs of volume depletion, CNS signs (e.g., decreased level of consciousness, headache, or abnormal eye signs such as proptosis, pupillary dilation, diplopia, papilloedema), outpatient treatment failure, presence of comorbid conditions that require supportive medical care and extremes of age [7]. Korle Bu Teaching Hospital is presently the third largest Hospital in Africa. Being the premier national referral centre in Ghana with a 2,000-bed capacity, the hospital attends to patients from its nearby communities as well as all over the country, especially the southern zone of the country. The objective of this retrospective study was to determine the incidence of such severe odontogenic infections over the past 5 years at the Korle-Bu Teaching Hospital. The details of which will guide strategies in management. This review forms part of preliminary studies by the Department of Oral & Maxillofacial surgery unit of the hospital and the University of Ghana School of Medicine & Dentistry on the epidemiology, management, economic cost and microbiology of odontogenic infections.

## Methods

A retrospective descriptive review was done, involving the consecutive inclusion of all patients with severe odontogenic infection requiring admission to the Maxillofacial unit in the period between 1^st^ August 2012 and 31^st^July 2017. For all patients with near-fatal odontogenic infections admitted, independent variables including age, sex, diagnosis, duration of hospital stay, National Health Insurance (NHIS) status, and outcome of management were obtained from patient records, entered into a computerized questionnaire, processed and analyzed using Stata statistical software (version 14). The incidence proportion, which is a fraction of the total number of severe cases seen per the total number of tooth-related infections were obtained for the respective years. The Department's annual reports for the past 5 years were also reviewed to determine the total hospital attendance to the Department.

## Results

**Figure 1:**
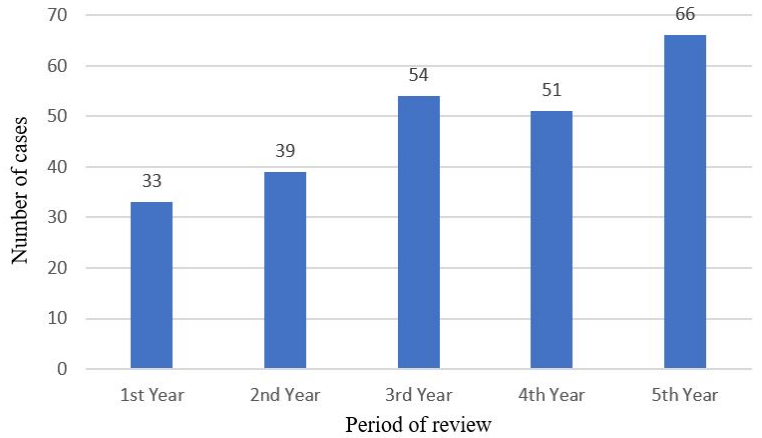
Annual admissions of near-fatal odontogenic infections

**Figure 2:**
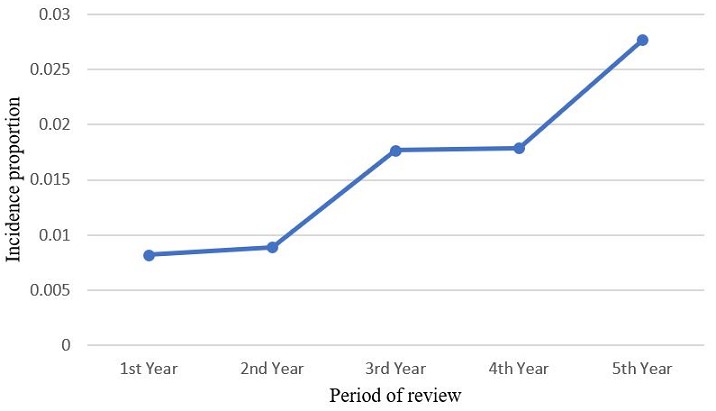
Incident proportions of severe odontogenic infection

**Figure 3:**
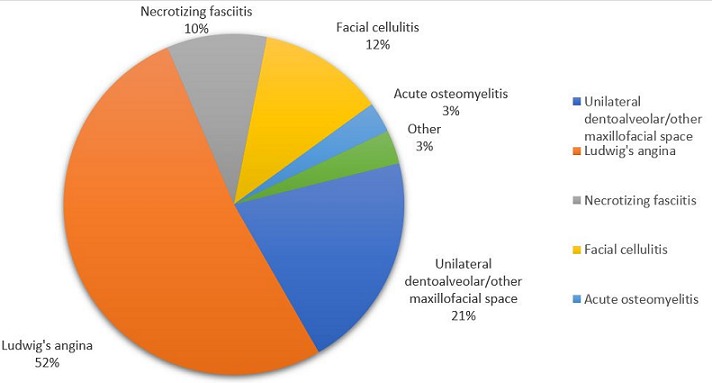
Distribution of admitting diagnoses

Annual records of diagnosed tooth-related infections totaled 4018, 3374, 3049, 2839 and 2384 patients for the respective years of review. The maxillofacial unit, for the past 5 years therefore recorded 15,664 cases of odontogenic infections, out of a total of 39,194 patients seen at the department in general. Of these, a total of 243 patients with near-fatal severe odontogenic infection, requiring admission were included in the survey for the period of the study, this representing 1.6% of all dento-alveolar infections. This consisted of 121 males and 122 females, representing a ratio of 1:1. With an average of 42.9 years (SD=16.6), the age ranged from 18 months to 91 years. The distribution of occurrence of severe odontogenic infection as shown in Figure 1 indicates a steady rise in admission of such cases. The incidence proportions of near-fatal odontogenic infection for the five-year study period were 8.2, 8.9, 17.7, 17.9 and 27.7 people per 1000 cases of tooth-related infections for the respective years (Figure 2). The incidence, however of odontogenic infections among patients attending the outpatient dental clinic of the hospital was 40.3%, while that of dentoalveolar abscess was 5.7%. The incidence of severe, near-fatal odontogenic infections among patients attending dental clinic of the hospital was 0.62%. Most (52%) of the patients presented with bilateral cellulitis of the submandibular, sublingual and submental spaces, characteristic of Ludwig's angina, while a proportional 3%, as shown in Figure 3, included other manifestations of odontogenic infections such as deep cervical and pharyngeal abscess, sepsis, intracranial abscess and infra orbital abscess. The mean hospital stay for the patients with severe odontogenic infection was 9.1 days (SD=5.9). Furthermore, the recorded deaths from the admitted cases were 14, representing a fatality rate of 5.8%. Of the admitted cases of severe odontogenic infections, 31.3% were not beneficiaries of the National Health Insurance scheme.

## Discussion

**Figure 4:**
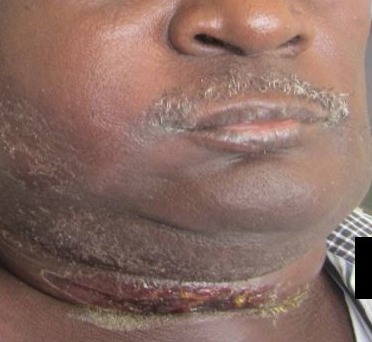
Severe odontogenic infection manifesting as Ludwig’s angina

**Figure 5:**
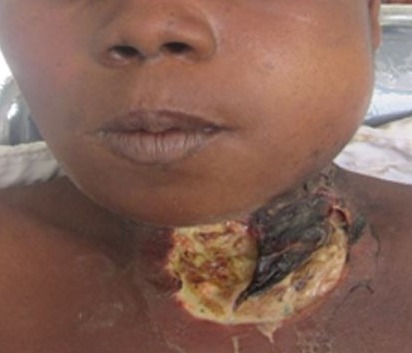
Necrotizing cervical fasciitis from a dentoalveolar infection

**Figure 6:**
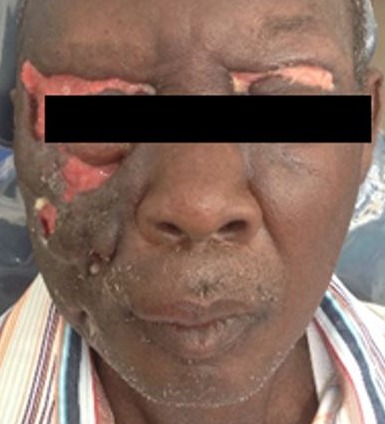
Orbital cellulitis resulting from a dentoalveolar infection

**Figure 7:**
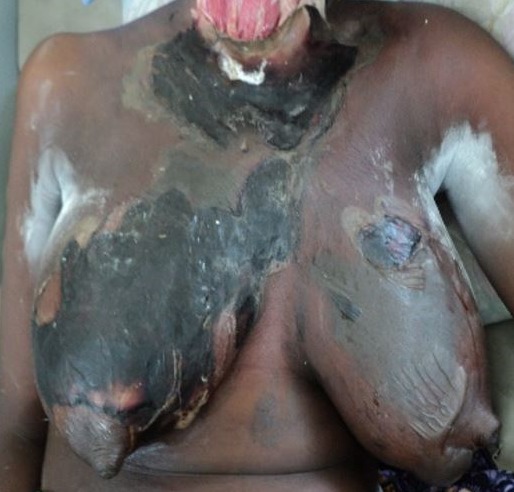
Descending necrotizing fasciitis resulting in bilateral breast abscess and anterior mediastinitis, secondary to a dentoalveolar infection

Odontogenic infections have for over the past century, been recognized as an important cause of morbidity and mortality [8], though the knowledge of the potential for tooth abscess to spread has been known since antiquity, mentioned in the London Bills of Mortality in the early 1600´s, as a leading cause of death [9]. Severe odontogenic infection and its complications are a sequelae to the breach of the pulp chamber by implicated bacteria. The pathophysiology of the condition has been very well described in literature [4, 5, 6, 10]. Though cases of odontogenic infections, in general, seem to have reduced over the years, findings of the incidence of severe odontogenic infections from this study do not favour the suggestion that its incidence is reducing in Ghana. The rise in new cases of severe odontogenic infections could be accounted for by either the increasing risk factors among the populace or by the relative attrition of Oral & Maxillofacial Surgeons in peripheral hospitals. In either case, the condition seems to persist as a pertinent clinical problem in Ghana that needs to be addressed. Though the intensity and criteria for admissions may vary across institutions and communities, the situation may not be peculiar to this part of the world alone. Scotland recorded 3,500 hospital admissions between 2000 and 2005 for acute dental infections while hospitals in England saw a doubling of admissions for surgical treatment of dental abscesses over a similar period [6]. Similarly, admissions in the United States relating to acute dental infections are estimated to occur at a rate of 1 per 2,600 of the population per year [11]. Though this study found a 0.62% incidence of severe dentoalveolar abscess requiring admissions among patients attending the hospital's dental clinic, studies in Nigeria found 6.4% of dental clinic attendance being because of dentoalveolar abscess [12]. In Australia, 9% of emergency dental presentations resulted in admissions [13]. The incidence and varying presentations of severe odontogenic infections should also reinforce the need for Clinicians, to exclude odontogenic causes of conditions such as facial cellulitis, deep neck abscesses and descending mediastinitis. It has also been reported that the incidence of deep neck space infection is significantly higher in patients with odontogenic abscess, compared with those without odontogenic abscess [1]. Typical of patient in Figure 4, most (52%) of the patients were admitted with the diagnosis of Ludwig's angina. Also, 10% of the patients had cervico-facial necrotizing fasciitits (Figure 5), with a significant proportion (3%) presenting with other complications including sepsis, intra-cranial abscess, periorbital abscess (Figure 6), anterior descending mediastinitis (Figure 7), and infratemporal abscess. This, together with the fatality rate of 5.8%, suggest that the patients presented late to the facility.

## Conclusion

This study highlights the importance of persisting severe, near-fatal odontogenic infections in Ghana. Its clinical complications may require a multidisciplinary approach in treatment. However, not only is there a need to assess the public, professional and institutional strategies to management, but for more evidence-based studies in our local setting to aid in management.

### What is known about this topic

- Odontogenic infections have the potential to spread to anatomical spaces, with severe odontogenic potentially leading to mortality;
- Odontogenic infections, in their severe forms are considered to be decreasing in occurrence.

### What this study adds

- The burden of severe odontogenic infection is not decreasing in Ghana. Extensive cervicofacial cellulitis and fasciitis from the condition is still prevalent, thus requiring health policy attention and further inquiry.

## Competing interests

The authors declare no competing interests.
